# Enhanced Stomatal Conductance Supports Photosynthesis in Wheat to Improved NH_4_^+^ Tolerance

**DOI:** 10.3390/plants13010086

**Published:** 2023-12-27

**Authors:** Jinling Hu, Qiaomei Zheng, Chaofeng Dong, Zhihui Liang, Zhongwei Tian, Tingbo Dai

**Affiliations:** Key Laboratory of Crop Physiology Ecology and Production Management of Ministry of Agriculture, Nanjing Agricultural University, Nanjing 210095, China; 2018201007@njau.edu.cn (J.H.); 2020201015@stu.njau.edu.cn (Q.Z.); 2021101015@stu.njau.edu.cn (C.D.); 2015101019@njau.edu.cn (Z.L.); zhwtian@njau.edu.cn (Z.T.)

**Keywords:** ammonium stress, photosynthesis, wheat, stomatal conductance, osmatic potential

## Abstract

The impact of ammonium (NH_4_^+^) stress on plant growth varies across species and cultivars, necessitating an in-depth exploration of the underlying response mechanisms. This study delves into elucidating the photosynthetic responses and differences in tolerance to NH_4_^+^ stress by investigating the effects on two wheat (*Triticum aestivum* L.) cultivars, Xumai25 (NH_4_^+^-less sensitive) and Yangmai20 (NH_4_^+^-sensitive). The cultivars were grown under hydroponic conditions with either sole ammonium nitrogen (NH_4_^+^, AN) or nitrate nitrogen (NO_3_^−^, NN) as the nitrogen source. NH_4_^+^ stress exerted a profound inhibitory effect on seedling growth and photosynthesis in wheat. However, these effects were less pronounced in Xumai25 than in Yangmai20. Dynamic photosynthetic analysis revealed that the suppression in photosynthesis was primarily attributed to stomatal limitation associated with a decrease in leaf water status and osmotic potential. Compared to Yangmai20, Xumai25 exhibited a significantly higher leaf K^+^ concentration and *TaAKT1* upregulation, leading to a stronger stomatal opening and, consequently, a better photosynthetic performance under NH_4_^+^ stress. In conclusion, our study suggested stomatal limitation as the primary factor restricting photosynthesis under NH_4_^+^ stress. Furthermore, we demonstrated that improved regulation of osmotic substances contributed to higher stomatal conductance and enhanced photosynthetic performance in Xumai25.

## 1. Introduction

It is well known that ammonium (NH_4_^+^), though a principal nitrogen source, can induce plant toxicity, particularly when used as the sole or predominant nitrogen source [1,2,3]. Several studies have investigated the manifestations of NH_4_^+^ stress in plants, including diminished biomass, elevated reactive oxygen species (ROS) production, disruptions in pH equilibrium, and ion regulation [2,4]. The sensitivity of plants to NH_4_^+^ stress is inherently variable, contingent upon factors such as species, genotypes, and soil conditions [1,4,5]. Though NH_4_^+^ assimilation heavily relies on the sugar generated during photosynthesis in the leaves, extant research has predominantly concentrated on the mechanisms underlying NH_4_^+^ uptake and assimilation in plant roots [6,7]. A discernible knowledge gap exists in how NH_4_^+^ stress affects photosynthesis, especially in susceptible plant species.

During photosynthesis, the diffusion of CO_2_ from the atmosphere to the chloroplast sites for carboxylation encounters multiple resistances within leaves. The regulatory mechanism underlying stomatal opening is well elucidated [8]. It comprises an initial influx of K^+^, which is later replaced by sucrose, culminating in a reduction of sucrose levels [9]. Recent investigations in rice plants have demonstrated that K^+^ deficiency can reduce stomatal conductance, restricting gas exchange [10]. Furthermore, NH_4_^+^ absorption has been shown to impede the uptake of other cations, such as K^+^ or Ca^2+^, and elevate ABA content [4,11]. Thus, NH_4_^+^ stress might instigate a reduction in stomatal conductance, adversely impacting photosynthesis.

While the regulation of stomatal conductance (*g_s_*) and transpiration by nitrogen nutrition is a well-established tenet in plant physiology, the impact of excess NH_4_^+^ on transpiration and water use efficiency (WUE) remains unclear. Intriguingly, distinct nitrogen forms can impart divergent effects on plant water uptake, this phenomenon is intricately linked to the nitrogen preference of various plant species [12]. Some studies posit that excessive NH_4_^+^ can inhibit plant water uptake [13] or induce symptoms akin to water deficit, termed “ammoniacal syndrome” [14]. Conversely, other studies argue that compared to NO_3_^−^ nutrition, NH_4_^+^ nutrition can further strengthen water stress resistance in rice via marked upregulation of AQP genes [12,15]. Thus, the influence of NH_4_^+^ stress on the water status of plants and its intricate relationship to stomatal opening and subsequent photosynthetic capacity remain unclear.

Chlorophyll fluorescence, closely linked to chlorophyll content, emerges as a crucial indicator of the photosynthetic response to stress [16]. NH_4_^+^ stress adversely impacts the chlorophyll content of plants, however, the degree of impact depends on the severity of stress and the plant species [17,18]. Previous studies have postulated that NH_4_^+^ stress can disrupt the stability of leaf membrane lipids, hindering electron transfer and consequently impeding photosynthesis [19]. In contrast, a previous study in *Arabidopsis* showed that NH_4_^+^ stress elevates mitochondrial ROS levels without significant impacts on photosynthesis [20]. Such divergent findings warrant the need to comprehensively elucidate the regulatory mechanisms governing chlorophyll content and chlorophyll fluorescence under NH_4_^+^ stress.

Wheat, a key part of global food production, is well known for its high susceptibility to NH_4_^+^ toxicity [21,22]. In wheat seedlings, NH_4_^+^ stress is manifested as stunted seedling growth and the onset of oxidative stress [21]. The pivotal role of nitrogen in the metabolism of photosynthetic pigments and the efficient functioning of the photosynthetic apparatus underscores its profound impact on overall plant growth and development, including in wheat. Despite the indispensable role of nitrogen in photosynthesis, the specific photosynthetic response of wheat seedlings to NH_4_^+^ stress remains unknown. The primary objective of this study was to delve into the mechanism underlying the tolerance and photosynthetic response of wheat seedlings to NH_4_^+^ stress. Here, we conducted a comparative analysis of two wheat cultivars: NH_4_^+^-less sensitive Xumai25 and NH_4_^+^-sensitive Yangmai20. We focused on growth responses, gas exchange, leaf water status, and adjustments in osmotic balance in both these cultivars in response to NH_4_^+^ stress.

## 2. Results

### 2.1. Plant Growth and Nitrogen Concentration

The impact of NH_4_^+^ stress (AN) on plant growth was evident from the significantly lower dry weight of AN-treated plants than NN-treated plants at 10 DAT, with a more pronounced decrease in Yangmai20 (25%) than Xumai25 (7%). Similarly, the AN-treated plants exhibited reduced leaf area, with a more pronounced reduction in Yangmai20 than Xumai25 (Table 1). In contrast, the AN-treated plants showed higher specific leaf weight (SLW) and nitrogen concentration than the NN-treated plants, with a more pronounced increase in Yangmai20.

### 2.2. Photosynthesis and Related Attributes

**Figure 1:**
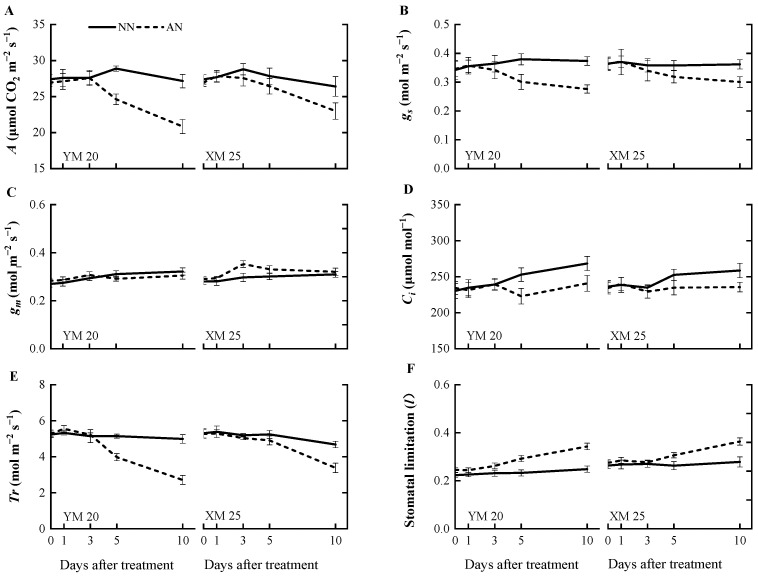
Effects of ammonium stress on (**A**) net photosynthetic rate (*A*), (**B**) stomatal conductance (*g_s_*), (**C**) leaf mesophyll conductance (*g_m_*), (**D**) sub-stomatal CO_2_ concentration (*C_i_*), (**E**) transpiration rate (*Tr*), and (**F**) stomatal limitation (*l*) of wheat seedlings at 0, 1, 3, 5, and 10 days after treatment. NN, nitrate treatment; AN, ammonium treatment. YM20, Yangmai20 (NH_4_^+^-sensitive); XM25, Xumai25 (NH_4_^+^-less sensitive).

**Figure 2:**
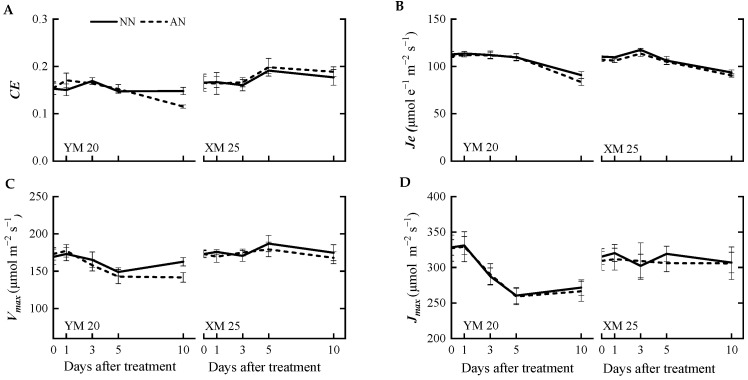
Effects of ammonium stress on the (**A**) carboxylation efficiency (*CE*), (**B**) electron transfer rate (*J_e_*), (**C**) maximum carboxylation rate (*V_max_*), and (**D**) the maximum electron transport rate (*J_max_*,) at 0, 1, 3, 5, and 10 days after treatment NN, nitrate treatment; AN, ammonium treatment. YM20, Yangmai20 (NH_4_^+^-sensitive); XM25, Xumai25 (NH_4_^+^-less sensitive).

The AN-treated plants exhibited a progressive decline in the net photosynthetic rate (*A*) (Figure 1A) from 1 to 10 DAT, with a more pronounced decline observed in NH_4_^+^-sensitive Yangmai20. At 5 DAT, the value of *A* was significantly reduced in Yangmai20 under NH_4_^+^ stress compared to NN-treated plants, but this phenomenon was not observed in Xumai25. Furthermore, AN significantly reduced stomatal conductance (*g_s_*), sub-stomatal CO_2_ concentration (*C_i_*), and transpiration rate (*Tr*) in Yangmai20 (Figure 1B,D,E), compared to the NN (17%, 11%, and 13%, respectively). In contrast, AN substantially increased stomatal limitation (*l*) in both cultivars, with consistently higher stomatal limitation, evidenced by lower *g_s_*, in Yangmai20 than in Xumai25 (Figure 1B,F). Additionally, AN did not significantly affect leaf mesophyll conductance (*g_m_*) (Figure 1C), carboxylation efficiency (*CE*), electron transfer rate (*J_e_*), maximum carboxylation rate (*V_max_*), or maximum electron transport rate (*J_max_*) in either cultivar (Figure 2A–D).

Furthermore, at 10 DAT, AN led to a significant decline in *CE*, *V_max_*, and *J_e_* of Yangmai20 but did not markedly impact the corresponding parameters in Xumai25 (Figure 2A–C).

### 2.3. Chlorophyll Content and Chlorophyll Fluorescence Parameters

**Figure 3:**
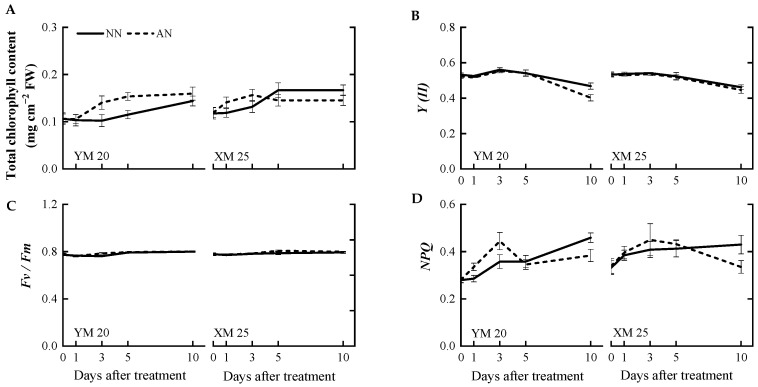
Effects of ammonium stress on (**A**) total chlorophyll content, (**B**) actual photochemical efficiency (*Y(II)*), (**C**) maximum quantum yield (*Fv/Fm*), and (**D**) non-photochemical quenching (*NPQ*) of wheat seedlings at 0, 1, 3, 5, and 10 days after treatment. NN, nitrate treatment; AN, ammonium treatment. YM20, Yangmai20 (NH_4_^+^-sensitive); XM25, Xumai25 (NH_4_^+^-less sensitive).

Changes in chlorophyll content and chlorophyll fluorescence were concurrently measured alongside photosynthetic parameters. The AN-treated Yangmai20 exhibited slightly elevated total chlorophyll content at 3 and 5 DAT, while this phenomenon was not observed in Xumai25. However, there was no significant difference in the total chlorophyll content of the AN- and NN-treated plants at 10 DAT (Figure 3A). Notably, no significant differences were observed in the actual photochemical efficiency (*Y(II*)) of AN- and NN-treated Xumai25 (Figure 3B–D). In contrast, the *Y(II)* value of AN-treated Yangmai20 was significantly lower than NN-treated Yangmai20 at 10 DAT (Figure 3B). Furthermore, no significant differences were observed in the maximum quantum yield (*Fv/Fm*) of the AN- and NN-treated plants. The non-photochemical quenching (*NPQ*) of AN-treated plants was significantly higher than NN-treated plants at 10 DAT.

### 2.4. Leaf Moisture Status 

**Figure 4:**
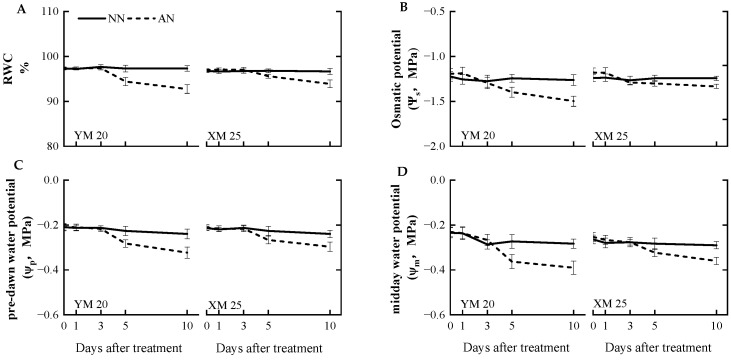
Effects of ammonium stress on the (**A**) relative water content (RWC), (**B**) leaf osmotic potential (ψs), (**C**) pre-dawn water potential (ψp), and (**D**) midday water potential (ψm) of wheat seedlings at 0, 1, 3, 5, and 10 days after treatment (DAT). NN, nitrate treatment; AN, ammonium treatment. YM20, Yangmai20 (NH_4_^+^-sensitive); XM25, Xumai25 (NH_4_^+^-less sensitive).

AN treatment adversely impacted the leaf moisture status compared to the NN treatment. At 5 DAT, AN-treated Yangmai20 exhibited significantly reduced relative water content (RWC), pre-dawn water potential (ψp), osmotic potential (ψs), and midday water potential (ψm) compared to NN-treated Yangmai20, while no significant differences were observed between AN- and NN-treated Xumai25. At 10 DAT, the AN-treated plants exhibited a decrease in RWC, ψs, ψp, and ψm, with a more pronounced decrease in Yangmai20 (Figure 4A–D).

### 2.5. Concentration of K^+^, Sucrose and ABA

**Figure 5:**
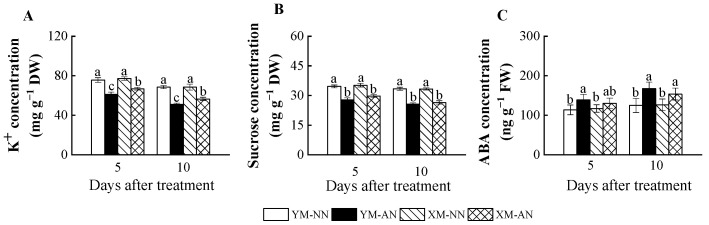
Effects of ammonium stress on (**A**) K^+^, (**B**) sucrose, and (**C**) ABA concentration of wheat seedlings at 5 and 10 days after treatment. Different letters indicate significant differences (*p* < 0.05) according to ANOVA. NN, nitrate treatment; AN, ammonium treatment. YM, Yangmai20 (NH_4_^+^-sensitive); XM, Xumai25 (NH_4_^+^-less sensitive).

AN treatment significantly impacted the parameters related to osmotic homeostasis and stomatal opening. All AN-treated plants exhibited consistently decreasing K^+^ and sucrose levels (Figure 5A,B). Although, Xumai25 exhibited substantially less pronounced K^+^ reduction than Yangmai20 (Figure 5A). In addition, AN-treated Yangmai20 exhibited higher ABA concentration in the leaves than NN-treated plants at 5 DAT, while no significant differences were observed between AN- and NN-treated Xumai25. At 10 DAT, ABA concentration was significantly increased in both cultivars under AN treatment (Figure 5C).

### 2.6. Relative Gene Expression

**Figure 6:**
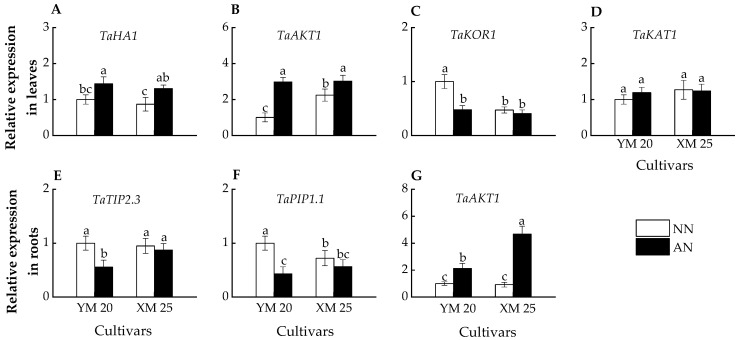
Effects of ammonium stress on the expressions of genes encoding K^+^ channel, water channel, and proton transport protein in leaves and roots of wheat seedlings at 5 days after treatment, including (**A**) *TaHA1*, (**B**) *TaAKT1*, (**C**) *TaKOR1*, (**D**) *TaKAT1*, (**E**) *TaTIP2.3*, (**F**) *TaPIP1.1*, (**G**) *TaAKT1*. Data are expressed as means of three biological replicates, and different letters indicate significant differences (*p* < 0.05) according to ANOVA. NN, nitrate treatment; AN, ammonium treatment. YM, Yangmai20 (NH_4_^+^-sensitive); XM, Xumai25 (NH_4_^+^-less sensitive).

In leaves, the AN-treated plants exhibited higher *TaHA1* and *TaAKT1* expression than NN-treated plants in both cultivars (Figure 6A,B). Furthermore, AN-treated Yangmai20 exhibited significantly lower *TaKOR1* expression than NN-treated Yangmai20, while no significant difference was observed between AN- and NN-treated Xumai25 (Figure 6C). However, the *TaKAT1* expressions of AN- and NN-treated Yangmai20 were comparable to AN- and NN-treated Xumai25, respectively (Figure 6D).

In roots, AN-treated Yangmai20 exhibited significantly lower *TaTIP2.3* and *TaPIP1.1* expression than NN-treated Yangmai20, while this phenomenon is not observed in Xumai25 (Figure 6E,F). Moreover, the AN-treated plants exhibited higher *TaAKT1* expression than NN-treated plants, with significantly higher expression in AN-treated Xumai25 than NN-treated Xumai25 (Figure 6G).

### 2.7. The Relationships between Photosynthetic Parameters and Physiological Traits with Nitrogen Forms

**Figure 7:**
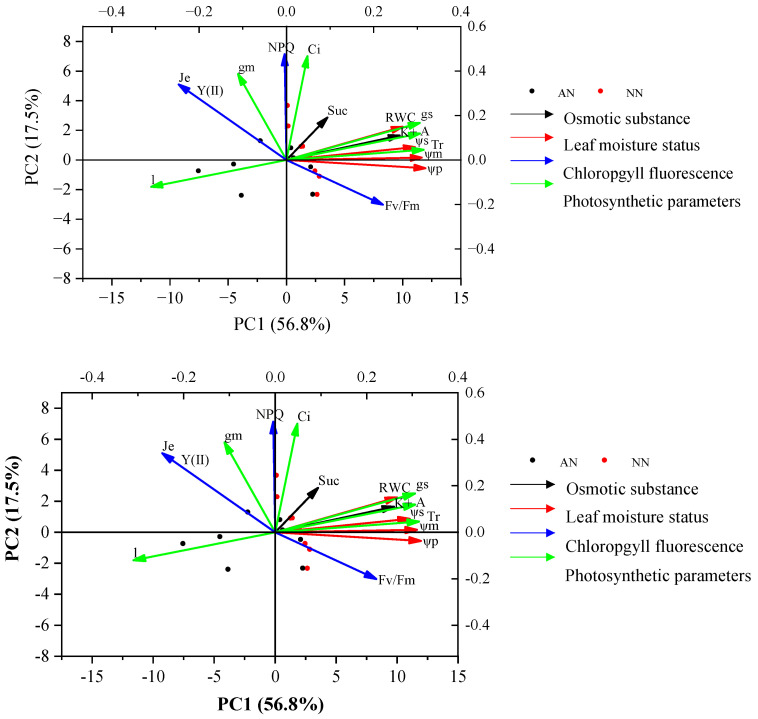
Principal component analysis (PCA) of photosynthetic parameters and other physiological traits of two wheat cultivars (Yangmai20 and Xumai25) under AN and NN treatments. NN, nitrate treatment; AN, ammonium treatment.

Principal component analysis (PCA) demonstrated that PC1 and PC2 explained 74.3% of the total trait variance for the two wheat cultivars under the two treatments (AN and NN) (Figure 7). The eigenvalue and cumulative contribution of PC1 and PC2 of photosynthetic parameter and physiological traits and their corresponding loading are shown in Supplementary Table S1. PC1 explained 56.8% of total variation and had a positive association with ψm, ψs, ψp, K^+^, *g_s_*, and *A*, and a negative association with *l*. Thus, PC1 tended to represent traits like leaf moisture status, osmotic substance, and photosynthetic parameters. Alternatively, PC2 explained 17.5% of the total variation and was positively associated with *NPQ* and *Y(II)*, representing the chlorophyll fluorescence. These results indicated that photosynthetic traits might be positively related to leaf moisture status and osmotic substance, and were negatively correlated to *l*.

## 3. Discussion

Diverse plant species and cultivars manifest distinctive responses to NH_4_^+^ stress, as demonstrated by studies on peas [23], tomatoes [24], and *Arabidopsis* [25]. Several studies have extensively reported the NH_4_^+^ sensitivity of wheat [21,26]. In this study, the deleterious impact of NH_4_^+^ stress on the biomass and photosynthesis of wheat seedlings (Table 1 and Figure 1A) is similar to the effects reported in other plant species [2,27]. Notably, upon NH_4_^+^ stress, the NH_4_^+^-less sensitive cultivar Xumai25 demonstrated a mitigated reduction in net photosynthetic rate (*A*) compared to the NH_4_^+^-sensitive cultivar Yangmai20, resulting in superior dry matter production (Table 1, Figure 1A).

### 3.1. Variation in Photosynthetic Response and Stomatal Limitation

Photosynthesis, intricately linked to NH_4_^+^ tolerance [28], involves limitation and biochemical limitations influencing photosynthetic capacity. Our findings indicated that Yangmai20 exhibited a notable reduction in *A*, *g_s_*, *C_i_*, and an increase in *l* (Figure 1A–D). However, parameters related to the photosynthetic dark reaction, such as *CE*, *J_e_*, *V_max_*, and *J_max_*, did not differ significantly between AN- and NN-treated plants (Figure 2A–D). Previous studies have demonstrated *l* to be a critical factor in reducing *A* when both *C_i_* and *g_s_* decrease [29,30]. Thus, in the present study, *l* might predominantly contribute to the reduced photosynthesis in wheat under NH_4_^+^ stress. Consistent with our findings, NH_4_^+^ stress has previously been found to induce stomatal closure in tomatoes [28], and reduction in rice [31] and wheat [32]. Notably, Xumai25 exhibited a less pronounced decrease in *g_s_* at 5 DAT, indicating its superior ability to maintain the open state of stomata, contributing to enhanced photosynthetic performance under NH_4_^+^ stress. Furthermore, at 10 DAT, the dark reaction parameters (*CE*, *V_max_*, and *J_e_*) significantly decreased in Yangmai20 under prolonged NH_4_^+^ stress (Figure 2A,C,D), illustrating a delayed response, which might be attributed to initial CO_2_ assimilation induced by reduced *g_s_* [33].

### 3.2. Leaf Mesophyll Conductance (g_m_) under NH_4_^+^ Stress

The impact of NH_4_^+^ on *g_m_* varies based on the plant species and severity of the stress. For instance, Liu et al. (2021) [34] found a significantly lower *g_m_* in female Populous cathayana plants after being supplied with NH_4_^+^ under salt stress than after being supplied with NO_3_^−^. In contrast, Li et al. (2012) [35] reported a significantly higher *g_m_* in NH_4_^+^- supplied rice than NO_3_^−^-supplied rice under drought stress. In the present study, however, we did not observe a significant difference in *g_m_* between AN- and NN-treated plants (Figure 1C). The observed variations in *g_m_* can be attributed to two factors. First, increased NH_4_^+^ assimilation products augmented the specific leaf weight (SLW) in both cultivars (Table 1), influencing CO_2_ partial pressure inside chloroplasts, which can increase *g_m_* [36]. Second, heightened leaf nitrogen content (Table 1) after NH_4_^+^ stress possibly led to increased cell wall thickness [37].

### 3.3. Chlorophyll Concentration and Fluorescence under NH_4_^+^ Stress

The impact of NH_4_^+^ stress on chlorophyll content varies based on stress severity, plant species, and cultivars [18]. For instance, several studies have reported a decrease in the chlorophyll content of *Arabidopsis* in the presence of high NH_4_^+^ levels [17,38]. In contrast, some studies have reported increased and decreased chlorophyll content after mild and severe NH_4_^+^ stress, respectively [18]. In the present study, the chlorophyll content in both cultivars was not markedly different between NN-treated and AN-treated plants (Figure 3A), suggesting that 5 mM NH_4_^+^ was considered a moderate stress level for wheat seedlings. Additionally, chlorophyll content is closely associated with the efficiency of electron transport and photosynthesis. For instance, Chen et al. (2023) [27] demonstrated that NH_4_^+^ exposure disrupts the electron transport chain in citrus plants. Interestingly, in this study, we did not observe any significant difference in *J_e_* as *A* decreased in Yangmai20 at 5 DAT and in Xumai25 at 10 DAT. Chlorophyll fluorescence measurements indicated no significant damage to the PSII reaction centers during the treatment period, as evident by the unchanged *Fv/Fm* in both cultivars (Figure 3C). The reduction in *Y(II)* in AN-treated Yangmai20 (Figure 3B) might be responsible for the decrease in *J_e_* (Figure 2B). In addition, the increase in *NPQ* in AN-treated plants was possibly related to photorespiration [39].

### 3.4. Decline in Stomatal Conductance and Osmotic Potential under NH_4_^+^ Stress

Several factors, such as stomatal morphology, distribution, and movement, influence *g_s_*, a pivotal determinant of photosynthesis [40]. The focus of the current investigation was the nuanced impact of NH_4_^+^ stress on *g_s_*, with specific attention to the intricate regulation of stomatal movement. We meticulously examined the newest fully expanded leaves before treatment initiation to circumvent potential confounding effects from stomatal morphology and distribution. Stomatal movement, primarily orchestrated by the changes in the turgor pressure and volume of the guard cells, is governed by major osmotic entities, such as K^+^ and sucrose [8]. Our findings revealed a marked reduction in the leaf osmotic potential (Ψs) of Yangmai20 since 5 DAT (Figure 4B), aligning with the decline in *g_s_*. This finding indicates a direct correlation between diminishing Ψs under NH_4_^+^ stress and the consequential reduction in stomatal aperture.

Recent research emphasizes the pivotal role of sucrose and K^+^ as primary solutes influencing the guard cell osmotic potential [8,9]. K^+^ deficiency has been shown to reduce leaf water potential and stomatal area in rice, resulting in a decline in photosynthesis [10]. In the current study, a notable reduction in leaf K^+^ concentration was observed for both wheat cultivars under NH_4_^+^ stress, with a more pronounced reduction in Yangmai20 (Figure 5A). Intriguingly, our study unveiled a higher *TaAKT1* upregulation under NH_4_^+^ stress in the roots of Xumai25 than Yangmai20 (Figure 6G), potentially contributing to the higher K^+^ concentration in Xumai25. Moreover, we observed a significant decrease in the sucrose level of both cultivars under NH_4_^+^ stress (Figure 1A), which potentially decreases osmotic potential. The decreased sucrose concentration is potentially linked to inhibited photosynthesis (Figure 1A) and increased carbon skeleton requirements due to ammonium assimilation in the roots [2]. Taken together, the reduction in K^+^ and sucrose levels under NH_4_^+^ stress led to a decrease in leaf osmotic potential, which in turn resulted in a decrease in *g_s_*. Xumai25, benefiting from its higher leaf K^+^ concentration, sustained an elevated Ψs under NH_4_^+^ stress, thus maintaining an enhanced *g_s_*.

Additionally, meticulous K^+^ flux regulation in guard cells, facilitated by inward (KAT) and outward rectifying K^+^ channels (KOR), also plays a crucial role [41]. Our study revealed a significant *TaKOR1* downregulation in Yangmai20 in response to NH_4_^+^ stress (Figure 6C), with no significant response in Xumai25. In contrast, no significant alteration in *TaKAT1* expression was observed in any of the cultivars (Figure 6D). This finding indicates that the high K^+^ concentration and influx in NH_4_^+^-less sensitive cultivars contributed to the consistently high osmotic potential under NH_4_^+^ stress. Additionally, both cultivars exhibited *TaHA1* upregulation (Figure 6A), indicating the crucial role of K^+^ flow [42] in NH_4_^+^ stress responses. Further, ABA has been demonstrated to activate anion channels in the plasma membrane of guard cells, leading to anion efflux and subsequent release of K^+^ from guard cells, resulting in stomatal closure under drought stress [42]. The elevated ABA level observed in our study (Figure 5C) further contributes to decreased *g_s_*. However, the nuanced interplay of NH_4_^+^ stress with ion channels governing osmotic potential in stomatal guard cells warrants further exploration. Our results provide an initial foundation for understanding this intricate relationship.

### 3.5. Water Uptake under NH_4_^+^ Stress

In the current study, the impact of NH_4_^+^ exposure on water uptake in the plants remained non-definitive. Some studies suggested that NH_4_^+^ nutrition enhances water absorption [15,43] while some studies proposed that exposure to NH_4_^+^ significantly inhibits water uptake in plants [13]. The present study observed a significant reduction in RWC and ψm in both cultivars, with a more pronounced effect in Yangmai20 (Figure 4A,D). Moreover, NH_4_^+^ stress prompted a substantial downregulation of *TaTIP2.3* and *TaPIP1.1* in Yangmai20 (Figure 6E,F). These findings indicate a more pronounced impact of NH_4_^+^ stress on water uptake in Yangmai20 than in Xumai25. Based on the existing literature, we postulate that this phenomenon might be attributed to two factors. First, the downregulation of AQPs under NH_4_^+^ stress to mitigate NH_3_/NH_4_^+^ absorption and transport, as some studies have reported that aquaporins (AQPs) can transport NH_3_/NH_4_^+^ [12,44]. Second, the cellular acidification induced by NH_4_^+^ stress [2], as evidenced by *TaHA1* upregulation (Figure 6A), adversely impacts the activity of AQPs [45].

## 4. Materials and Methods

### 4.1. Plant Materials and Experimental Design

Two wheat cultivars, Xumai25 (NH_4_^+^-less sensitive) and Yangmai20 (NH_4_^+^-sensitive), were subjected to hydroponic experiments. The seeds were surface-sterilized using a 10% (*v*/*v*) H_2_O_2_ solution for 15 min and subsequently rinsed thoroughly with sterile distilled water. Subsequently, the seeds were germinated under dark conditions in Petri dishes until the seed bud length reached approximately 1 cm (typically 36 h). Then, the seedlings were transplanted into water-filled opaque plastic containers (45 cm × 32 cm × 25 cm, volume 36 L). When the seedlings reached the two-leaf stage, they were transferred to a modified 50% Hoagland nutrient solution and grown until they reached the four-leaf stage. After this pre-treatment, the seedlings were divided into two groups, each group comprising seedlings from both cultivars. One group was treated with nitrate nitrogen (NN, 5 mM NO_3_^−^-N) nutrient solution and the other with NH_4_^+^ nitrogen (AN, 5 mM NH_4_^+^-N) nutrient solution. The macronutrient compositions of both treatment solutions are detailed in Supplementary Table S2. The micronutrient composition was kept consistent in both treatment solutions, as described previously [21]. The solutions were refreshed every three days to ensure a consistent nitrogen supply in each solution and were continuously aerated to prevent anoxia. The pH of each solution was adjusted daily to 5.8 using either 0.1 mM H_2_SO_4_ or 0.1 mM NaOH. The entire experiment was conducted in a controlled greenhouse environment with a 16 h/8 h light/dark cycle and temperature maintained at 18 °C during the day and 8.5 °C at night. The light intensity and relative air humidity in the greenhouse were set at 400 µmol m^−2^ s^−1^ and 60%, respectively. We adopted a completely randomized block design, and each experiment was replicated three times. Each replication consisted of 3 containers, and each container housed 60 plants.

The random seedlings (belonging to both cultivars) from both groups were collected at 0, 1, 3, 5, and 10 days after treatment (DAT). The fourth leaf (the newest fully expanded leaf before treatment) was used for the measurement of photosynthetic, fluorescence, and leaf water status parameters. The fourth leaves, other leaves, stems, and roots of the collected seedlings were separated and divided into two segments. One segment was oven-dried at 105 °C for 20 min, followed by drying at 85 °C, to determine the dry weight and nitrogen concentration measurements. The other segment was promptly frozen in liquid nitrogen and stored at −80 °C for subsequent analyses.

### 4.2. Gas Exchange Measurements

Gas exchange measurements were constructed using a gas exchange system (Li-Cor 6800, Li-Cor Inc. Lincoln, NE, USA) equipped with a standard chamber (2 cm^2^) and a Multiphase Flash Fluorometer (6800-01F). The parameters were set as follows: leaf temperature, 25.0 ± 0.5 °C; steady-state photosynthetic photon flux density (PPFD), 1200 μmol photons m^−2^ s^−1^; vapor pressure deficit (VPD), 1.1 ± 0.05 kPa; and relative humidity, 55–65%. Leaves were introduced into the chamber at a reference CO_2_ concentration of 400 μmol mol^−1^ for 10 min for stabilization before measurement. Parameters including net photosynthetic rate (*A*), sub-stomatal CO_2_ concentration (*C_i_*), stomatal conductance (*g_s_*), transpiration rate (*Tr*), and electron transport rate (*J_e_*) were recorded after stabilization. 

For the A–Ci curves, the reference CO_2_ concentration was systematically adjusted to the following levels: 400, 200, 150, 100, 50, 400, 400, 600, 800, 1000, 1200, and 1500 μmol mol^−1^. Data were recorded, following a stabilization period of 2–3 min at each level. Six leaves from each treatment group were selected for this experiment. Carboxylation efficiency (*CE*) was calculated as the initial slope of the A–Ci curves when *C_i_* was <200 μmol mol^−1^ [46]. Parameters such as maximum carboxylation rate (*V_max_*), RuBP regeneration (*J_max_*), mesophyll conductance (*g_m_*), and stomatal limitation (*l*) were determined using a modified equation derived from the works of S. P. Long and C. J. Bernacchi [47] and Li et al. [46]. 

The photosynthetic rate (*A*) was expressed mathematically as:

*A* = *v_c_* − 0.5*v_o_* − Rd = *v_c_* (1 − Γ*/*C_i_*) − Rd (1)

Here, *v_c_* is the Rubisco carboxylation rate, *v_o_* is the Rubisco oxygenation rate, Rd is the mitochondrial respiration rate in the light, and Γ* is the CO_2_ compensation point related to the *C_i_* [46]. Here, *v_c_* is determined by the minimum of three potential rates: potential Rubisco carboxylation rate, RuBP regeneration rate, and triose phosphate utilization (TPU) rate, (*w_c_*, *w_j_*, and *w_p_*, respectively):

*v_c_* = min (*w_c_*, *w_j_*, *w_p_*) (2)

Also:

*w_c_* = (*V_max_* × *C_i_*)/(*C_i_* + *Kc* × (1 + *O*/*Ko*)) (3)

*w_j_* = (*J_max_* ×*C_i_*)/(4.5 *C_i_* + 10.5 Γ*) (4)

Here, *O* is the O_2_ concentration (210 mmol mol^−1^). Furthermore,

*Kc* = exp^(38.05 − 79.43/(*R* × (*T* + 273.15)))^ (5)

*Ko* = exp^(20.30 − 36.38/(*R* × (*T* + 273.15)))^ (6)

Here, *Kc* and *Ko* are the Michaelis constants of carboxylation and oxygenation, respectively. T is the leaf temperature (°C). 

The A–Ci curve comprises three phases. In the first phase, Rubisco is limiting and Je increases with the increase in *C_i_*. Thus Equation (3) can be fitted to Equations (1) and (2) as follows:

*A* = *V_max_* × *C_i_* × (1 − Γ*/*C_i_*)/(*C_i_* + *Kc* × (1 + *O*/*Ko*)) − Rd (7)

Setting f as a variable changing with *C_i_*:

f = *C_i_* × (1 − Γ*/*C_i_*)/(*C_i_* + *Kc* × (1 + *O*/*Ko*)) (8)

Equation (8) can then be rewritten as follows:

*A* = *V_max_* × *f* − Rd (9)

Thus, *A* can be plotted as a linear function of *f*, where *V_max_* is the slope and Rd is the intercept. 

In the second phase, RuBP regeneration is limiting and *J_e_* remains constant with increasing *C_i_*. Thus, Equation (4) can be fitted to Equations (1) and (2) as follows:

*A* = *J_max_* × *C_i_* × (1 − Γ*/*C_i_*)/(4.5 *C_i_* + 10.5 Γ*) − Rd (10)

Setting *g* as a variable changing with *C_i_*:

*g* = *C_i_* × (1 − Γ*/*C_i_*)/(4.5 *C_i_* + 10.5 Γ*) (11)

Equation (11) can then be written as follows:

*A* = *J_max_* × *g* − Rd (12)

Thus, *A* can be plotted as a linear function of *g*, where *J_max_* is the slope and Rd is the intercept. 

Stomatal limitation (*l*) was calculated as follows:

*l* = (A″ − A′)/A″ (13)

Here, A′ is the net photosynthetic rate at the ambient atmospheric CO_2_ concentration (400 μmol mol^−1^) and A″ is the net photosynthetic rate when *C_i_* is equal to ambient atmospheric CO_2_ concentration (C_i_ = 400 μmol mol^−1^).

The leaf mesophyll conductance (*g_m_*) was calculated using the constant J method [47,48]:

*g_m_* = *A*/(*C_i_* − Γ* (*J_e_* + 8(*A*+ Rd))/(*J_e_* − 4(*A*+ Rd))) (14)

Here, *J_e_* is the electron transport rate.

### 4.3. Leaf Area and Chlorophyll Fluorescence Measurements

Plant leaf areas were measured using an LI-3100 AREA METER (Li-Cor, Inc., Lincoln, NE, USA). 

After the photosynthetic measurements, the fluorescence characteristics of the fourth leaves were determined using a PAM-2500 portable chlorophyll fluorescence apparatus (PAM-2500, Walz, Germany). The leaves were dark-adapted for 30 min before measurements. The minimum fluorescence, Fo, and the maximal fluorescence yield, Fm, were recorded after executing a saturation pulse. Then the actinic light (497 μmol photons m^−2^ s^−1^) was opened, and the saturation pulses were generated. The initial fluorescence Fo’, the maximum fluorescence Fm’, and the steady fluorescence Ft were recorded during this progress. The maximum quantum yield of the PSII reaction center (*Fv/Fm*), the actual photochemical efficiency (*Y(II)*), and the non-photochemical quenching (*NPQ*) were calculated using a modified equation developed by Christof Klughammer (2008) [49]. The equation is as follows:

*Fv/Fm* = (Fm − Fo)/Fm (15)

*Y(II)* = (Fm′ − F)/Fm′ (16)

*NPQ* = Fm/Fm′ − 1 (17)

### 4.4. Leaf Water Status

The pre-dawn water potential (ψp) and midday water potential (ψm) of the fourth leaf were determined during the pre-dawn period (between 04:00 and 05:00) and noon (between 12:00 and 13:00), respectively, using a Model 1505D-EXP Pressure Chamber Instrument (1505D-EXP, Decagon, Albany, OR, USA). Briefly, the leaf veins were vertically cut using a sharp blade and the leaf veins were immediately placed into the pressure chamber. The water potential was recorded as the pressure at which blisters emerged on the cross-section of the leaf.

The osmotic potential (Ψs) of the fourth leaf was determined using a vapor pressure osmometer (Wescor 5600, Wescor Inc., Logan, UT, USA) at 25 °C. Briefly, the leaf was flash-frozen in liquid nitrogen at 9:00–10:00 a.m. Then, cell sap was extracted through maceration and filtration using fine nylon mesh and a syringe. The osmotic potential was then calculated from the instrument, as per the manufacturer’s instructions.

The leaf relative water content (RWC) of the fourth leaf was measured using a previously described protocol [50]. Briefly, the leaves were weighed immediately after harvest and the weight was recorded as Wf. Then, the leaves were immersed in distilled water for 12 h and weighed to obtain the saturated fresh weight (Wt). Subsequently, the leaves were dried to a constant weight in an oven at 85 °C and weighed to obtain the dry weight (Wd). The RWC was calculated using the formula: (Wf − Wd) / (Wt − Wd) × 100%.

### 4.5. Determination of N, K+ and Sucrose Concentration

Fresh fourth leaves were freeze-dried and then ground into a powder.

For N and K concentration analyses, approximately 0.1 g of the sample powder was mixed with 5 mL of H_2_SO_4_. The resulting mixture was heated to 200 °C until a clear solution was obtained. Subsequently, the reaction was terminated by adding H_2_O_2_. The resulting solutions were then analyzed using inductively coupled plasma optical emission spectrometry (ICP-OES, Optima 8000, Perkin Elmer, Waltham, MA, USA).

The sucrose concentration was determined using the resorcinol method as described by Zeng et al. (2014) [51]. Briefly, 0.1 g of the sample powder was weighed and extracted with a sugar extraction solution. The mixture was centrifuged (15,000× *g*, 15 min), and the supernatant was collected. Next, 0.3 mL of the supernatant was mixed with 0.1 mL of 2 M NaOH, and the solution was incubated at 95 °C for 10 min. Next, 1 mL of 0.1 M resorcinol and 3.5 mL of 10 M HCl were added to the mixture, and the solution was incubated at 80 °C for 60 min. The OD of the solution was measured at 500 nm using UV/VIS spectrophotometer (Pharmacia, Cambridge, UK). The sucrose concentration was calculated using the standard curve.

### 4.6. Determination of ABA Concentration

ABA concentration was determined using the method described by Greco et al. (2012) [52], with some modifications. Briefly, approximately 0.5 g of the fresh leaf sample was weighed and added to a pre-cooled mortar with 5 mL of 50% chromatographic grade methanol (*v*/*v*) and ground into a slurry in an ice bath. The slurry was then extracted at 4 °C in the dark for 12 h. Afterward, the mixture was centrifuged at 10,000 r/min for 10 min at 4 °C, and the supernatant was collected and stored in a refrigerator. The residue from the first extraction was subjected to two more extractions. For the second extraction, 4 mL pre-cooled 80% chromatographic grade methanol was added to the residue, and the mixture was again extracted for 12 h, followed by centrifugation. The same progress was repeated for the third extraction using 2 mL of pre-cooled 100% chromatographic grade methanol. All supernatants from the three extractions were combined. To adsorb phenols and pigments, PVPP (0.2 g/g FW) was added to the supernatants, which were then shaken at 4 °C for 60 min and centrifuged as mentioned above. The resulting supernatant was slowly passed through a prepared C18 column, and the effluent was collected and freeze-dried in the dark. After freeze-drying, the sample was dissolved in 3 mL of 50% chromatographic grade methanol, filtered through a 0.22 μm membrane, and finally injected into an ultra-performance liquid chromatography (UPLC) system (ACQUITY UPLC H-Class system, Waters, Milford, MA, USA) for analysis. The UPLC analysis was performed using an ACQUITY UPLC HSS T3 column (100 mm × 2.1 mm × 1.8 μm, Waters).

### 4.7. RT-PCR Analysis

Total RNA was extracted from root and leaf samples using TRIzol reagent (Vazyme Bio, Nanjing, China). The HiScript III Q RT SuperMix (Vazyme Bio, Nanjing, China) was used for cDNA synthesis as per the manufacturer’s instructions. The cDNA samples were then diluted 5-fold and subjected to qPCR analysis. qRT-PCR was conducted using the CFX Connect Real-Time PCR Detection System (Bio-Rad, Hercules, CA, USA) with ChamQ SYBR qPCR Master Mix (Vazyme Bio, China).

The primer sequence for *TaHa1* was sourced from Jiang et al. [53]. The primers for aquaporins (*TaTIP2.3*, and *TaPIP1.1*) were sourced from Wu et al. [54]. The primers for potassium transport channel (AKT1) were designed using Primer 5 software. The primers for potassium ion inflow channel (KAT1), and potassium ion outflow channel (KOR1) were sourced from the work of Yang et al. [55]. ACT and ADP genes were used as internal references. All primer sequence is listed in Supplementary Table S3. Relative expression levels were determined using a previously described method [56].

### 4.8. Statistical Analysis

Physiological data derived from both dry and fresh samples were calculated using three biological replicates, while the data pertaining to photosynthetic, fluorescence, and water status parameters of the leaves were derived from six leaf replicates. Analysis of variance (ANOVA), followed by the Tukey HSD test was used for multiple comparisons. SPSS 26 (IBM, Armonk, NY, US) was used for all statistical analyses. Graphs and tables were generated using Origin 2021 software (OriginLab, Northampton, MA, USA) and Microsoft Excel.

## 5. Conclusions

In conclusion, our study showed that 5 mM NH_4_^+^ stress significantly disrupted the crucial photosynthetic processes in wheat plants. Our findings highlight the predominant stomatal limitation in photosynthesis under NH_4_^+^ stress conditions. In this process, K^+^ mediated Ψs decrease played a vital role in the decline of *g_s_*. In addition, the NH_4_^+^-less sensitive cultivar exhibited a robust ability to maintain osmotic homeostasis, resulting in higher *g_s_* and improved photosynthetic performance and growth under NH_4_^+^ stress. Further studies are still needed to elucidate how high NH_4_^+^ concentrations might impact, at the molecular and electrophysiological levels, the stomatal opening in higher plants.

## Figures and Tables

**Table 1:** Effects of ammonium stress on dry weight, leaf area, leaf nitrogen concentration, and specific leaf weight of wheat seedling at 10 days after treatment.

Cultivar	Treatment	Plant Dry Weight (mg plant^−1^)	Plant Leaf Area (cm^2^ plant^−1^)	Leaf Nitrogen Concentration (mg g^−1^)	Specific Leaf Weight of 4th Leaf (mg cm^−2^ DW)
Yangmai20	NN	1266 ± 39.7 a	100 ± 4.6 a	69 ± 1.9 b	0.30 ± 0.008 b
	AN	980 ± 31.2 c	82 ± 5.4 c	77 ± 2.5 a	0.38 ± 0.017 a
Xumai25	NN	1128 ± 50.3 b	97 ± 4.7 ab	65 ± 3.1 b	0.29 ± 0.013 b
	AN	1033 ± 50.2 c	86 ± 3.4 bc	75 ± 2.2 a	0.37 ± 0.006 a

Note: NN, nitrate treatment; AN, ammonium treatment. Data are means ± standard deviation (SD) of three biological replicates, and different letters indicate significant differences (*p* < 0.05) according to ANOVA.

## Data Availability

The data presented in this study are available on request from the corresponding author. The data are not publicly available due to we have an in-depth research project on this topic in the future.

## Supplementary Materials

The following supporting information can be downloaded at: https://www.mdpi.com/article/10.3390/plants13010086/s1. Table S1. Eigenvalue and cumulative contribution of PCs of photosynthetic parameter and their corresponding loading. Table S2. The concentration and components of macronutrients in both treatments. Table S3. The primer sequence in this study.
