# Supplementary material for: Enhanced Stomatal Conductance Supports Photosynthesis in Wheat to Improved NH4+ Tolerance
Source: Plants (Basel). 2023 Dec 27;13(1):86. doi: 10.3390/plants13010086 (PMC10780695; doi:10.3390/plants13010086)
Supplement: Supplementary file 1 [file plants-13-00086-s001.zip › plants-2754458-supplementary.pdf]

Supplementary Material

Table S1 Eigenvalue and cumulative contribution of PCs of photosynthetic parameter and their corresponding loading

PCs	Eigenvalue	Variance contribution rate %	Cumulative contribution rate %
1	9.91848	56.80025	56.80025
2	2.81164	17.49358	74.29384
3	1.77146	10.96433	85.25817

Photosynthesis and related parameters		Loading	
		PC1	PC2
Osmotic substances	Suc	0.09407	0.19112
	K^+	0.25998	0.11004
Leaf moisture status	RWC	0.26668	0.14955
	ψ_p	0.31871	-0.03719
	ψ_s	0.29453	0.05814
	ψ_m	0.31042	0.01048
Photosynthesis	*Tr*	0.31471	0.04608
	l	-0.30989	-0.12071
	g_m	-0.11068	0.38633
	Ci	0.04836	0.46685
	A	0.3065	0.11886
	g_s	0.30575	0.16694
Chlorophyll fluorescence	*Je*	-0.24672	0.33996
	Y(II)	-0.24672	0.33996
	NPQ	-0.004	0.47595
	Fv/Fm	0.22116	-0.20142

Table S2 The concentration and components of macronutrients in both treatments

Treatment	N (5 mM)	K (3 mM)	Ca (1.5 mM)	Mg (1 mM)	P (1 mM)
AN	$(NH_4)_2SO_4$	KH_2PO_4 / K_2SO_4	$CaCl_2$ / $CaSO_4$	$MgSO_4$	KH_2PO_4
NN	$Ca(NO_3)_2$ / KNO_3	KH_2PO_4 / KNO_3	$Ca(NO_3)_2$	$MgSO_4$	KH_2PO_4

Note: AN, ammonium stress treatment; NN, nitrite treatment.

Table S3. The primer sequence in this study

Gene name	Primer sequence (5' → 3')	Primer sequence (3' → 5')
TaHA1	CAACGACAAGAGCAGCTACC	GATGGTGTCGATGTCAAGGC
TaKAT1	TTCTGGGATGGGCTTCACAT	CCAGAATCTGTAGCGGGGAT
TaKOR1	CAAGGCAGTGCAGTTGATCA	TCTCCTCTTGACCGTCTTCG
TaTIP2.3	GCCATTCTAAGCCACCAT	ATGAACTCGGCGATGTAG
TaPIP1.2	TCCTCTGTCTGAACTTGA	GAATACTACTGCTACCACAT
TaTIP1:1	TATGAGGCGTTAGTTACC	CTGCTCCAATCATTCAAC
TaAKT1	CCCTACGACCGCCGATAC	ATTTCTGATGGGATGGTGGAG
ACT	CAGCAACTGGGATGATATGG	ATTTCGCTTTCAGCAGTGGT
ADP	GAGATGCGGATCCTGATGGT	CCCCGAGCTTGAGCTTGTAG
